# Mobility as a predictor of all-cause mortality in older men and women: 11.8 year follow-up in the Tromsø study

**DOI:** 10.1186/s12913-016-1950-0

**Published:** 2017-01-10

**Authors:** Astrid Bergland, Lone Jørgensen, Nina Emaus, Bjørn Heine Strand

**Affiliations:** 1 Department of Physiotherapy, Oslo and Akershus University College of Applied Sciences, Faculty of Health Sciences, Pilestredet, P.O. Box 4 St. Olavs plass, 0130 Oslo, Norway; 2 Department of Health and Care Sciences, UiT The Arctic University of Norway, 9037 Tromsø, Norway; 3 Department of Clinical Therapeutic Services, University Hospital of North Norway, Tromsø, Norway; 4 Department on ageing, Norwegian Institute of Public Health, Oslo, Norway; 5 Department of Community Medicine, Institute of Health and Society, University of Oslo, Oslo, Norway; 6 Norwegian National Advisory Unit on Ageing and Health, Tønsberg, Norway

**Keywords:** Mortality, Mobility, Self-rated health, Predictors

## Abstract

**Background:**

Disability in older adults is associated with loss of independence, institutionalization, and death. The aim of this study was to study the association between the Timed Up and Go (TUG) test and all-cause mortality in a population-based sample of older men and women.

**Methods:**

Our study population was home dwellers aged 65 and above, who participated in the fifth wave of the Tromsø study. This study included the TUG test and a range of lifestyle and mortality predictors. Participants were linked to the Cause of Death Registry and followed up for mortality for a maximum of 11.8 years. Cox regression was used to investigate the association between TUG and total mortality.

**Results:**

Mean TUG score was 12.6 s, and men performed better than women. The oldest participants had poorer TUG score compared to younger participants, increasing 0.25 s per year. There was a significant association between TUG and all-cause mortality, and the association was equally strong in men and women. Across the TUG-score categories, from quickest fifth to slowest fifth, the mortality increased in a step-wise fashion. Compared to the quickest fifth, the slowest fifth had hazard ratio (HR) of 1.79 (95% confidence interval (CI) 1.33, 2.42) in a model adjusted for age and gender. For each standard deviation TUG-score the increase in HR was 1.23 (95% CI 1.14, 1.33). The association between the TUG score and mortality remained significant after adjusting for self-reported health, body mass index, smoking and education.

**Conclusions:**

A significant association between the TUG score and mortality was observed in both men and women. Identifying older people with poor TUG may aid in identifying those at risk and thus targeted interventions may be applied.

## Background

Disability in older adults is associated with loss of independence, institutionalization, and death [1]. Even a small reduction in disability may translate into large healthcare savings and improvements in the physical, emotional, and social well-being of older adults [2]. Population aging is associated with an increase in the number of people who are disabled. This increase presents a challenge for society because elderly persons disabled in one or more domains of life are hospitalized more often and need more medical and long-term care [3–7], and face a higher mortality rate than nondisabled persons [8–15].

Difficulties in mobility are often the first sign of functional decline, and may indicate that a person could benefit from preventive actions [16]. Maintaining independent mobility is an important goal, especially for old women who are at a greater risk for functional decline and disability than older men [17]. Furthermore, mobility, or the ability to move about independently and safely in one’s environment, is fundamental to independent living and to the quality of life [18–20]. Walking is the fundamental mobility task for human life and is a complex neuromotor activity [21]. With advancing age, however, maintaining mobility and walking capability may be jeopardised by the increasing risk of physical and sensory impairments [22]. Medical or health conditions or advanced aging may disturb these subsystems causing slow walking, and slowing of movement with age appears to be a universal biological phenomena [23]. It is documented that persons walking slower than one meter per second are likely to be limited in energy needed for self-care, and persons walking faster than one meter per second may be expected to have the capacity to perform household activities [23]. Mobility is frequently measured through self- reports such as the ability to walk 400 m [24–27]. Moreover, longitudinal predictors of mobility decline have been documented including; increasing age [1, 28–30], number of morbidities [31], poor self-rated health [31], obesity [32], reduced leg strength [27, 33, 34], and psychosocial factors [31].

The “Timed Up and Go” (TUG) test is recommended [35] for screening frail persons and it can be carried out at home [36, 37]. The TUG test is found to mirror the physiological changes that occur with aging and physical inactivity [38] and reflects physical performance during every-day life [39]. Previous research have shown that TUG scores increase with increasing age [40]. The TUG test has been reported to yield high; interrater reliability, intrarater reliability and test- retest reliability [41].

Most studies have focused on the association between TUG score and falls [35, 42–45]. Fewer studies have investigated the predictive validity of TUG for subsequent onset of ADL disability [36, 45–47], frailty [48], fear of falling [49], hospitalization [45], decline in global health [45], and mortality [48]. Predictors of mortality in older populations are known to be somewhat different in men and women [50]. For example, Tiainen et al. [50] provided evidence that difficulties in daily activities and poor self-reported mobility are stronger predictors of mortality in men than women. To our knowledge only two community-based studies [51, 52] have investigated the relationship between TUG score and mortality, and only one of these included both genders.

Our aim was to study the association between TUG and all-cause mortality during 11.8 years follow-up in a population-based sample of older men and women aged 65 years and older at baseline, taking into account a range of lifestyle and health related factors.

## Methods

Participants were men and women in the fifth Tromsø study, phase 2 [53] in 2001–2002, aged 65 years or older at baseline (the majority being 75–79 years). The participation rate in this phase 2 study was 76%, and for our age group 1005 participated in the TUG test. Study participants were linked to the Cause of Death Registry and followed up for mortality until 01.01.2013. Mean follow-up time was 9.1 years (SD 3.2) and maximum follow-up time was 11.8 years. Reporting of data results was made in accordance to the STROBE statement checklist (see http://strobe-statement.org/index.php?id=available-checklists).

### Timed up and go

TUG was measured as the time (in seconds) it takes for the study participant to rise from a chair (with armrests), walk three meters quickly but safely, turn and walk back to the chair and sit down. A walking aid was allowed if needed [54]. For each combination of gender and ages 65–69 years, 70–79 years, and 80–88 years, participants were grouped in five equally sized groups based on their TUG-score. These age and gender specific TUG quintiles were used as the main exposure in our study. In addition, standardized TUG scores (z-scores) were created to have mean of zero and a standard deviation of one, and this was done for the similar gender and age group combinations as for the quintiles.

### Covariates at baseline

A priori we identified a small set of potential key confounders- that is, factors that have previously been shown to associate with both mobility and mortality. Height and weight were measured by trained personnel in The Tromsø study, and body mass index (BMI) was calculated as weight in kilograms divided by height in meters squared (kg/m^2^) and grouped into five groups: < 20 kg/m^2^, 20–24.9 kg/m^2^, 25–26.9 kg/m^2^, 27–29.9 kg/m^2^, 30+ kg/m^2^. Smoking was self-reported in three groups as current, previous, or never-smoker. Education level was based on years of education grouped as low (7 or less years), middle (8–12 years) or high (13 or more years). Self-reported current health status was categorized as: poor, not so good, good or very good.

### Statistics

Cox regression was used to investigate the association between TUG score at baseline and total mortality during follow-up. Attained age was used as time scale in the regression models, and participants who emigrated during followed up were censored at date of emigration. Analyses were run separately for men and women, as well as collapsed over genders (with the inclusion of an interaction term TUG by gender). First, analyses were adjusted for gender (age is already finely adjusted for because we use attained age as timescale). Secondly, we adjusted for BMI, self-reported health status, smoking and education. All analyses were performed on the sample with non-missing values for all the covariates (*N* = 864, 86% of full study sample). The proportional hazards assumption was checked on the basis of analysis of Schoenfeld residuals, and by graphical inspection of the estimated hazard function (on log-scale) on the Y-axis and analysis time on the X-axis. No violations of proportionality were observed (results not shown). Stata 13 was used for the analyses.

## Results

The participants had a mean age 76.7 years (SD = 2.8, range = 65-88) and 56.4% were women (Table 2). The mean TUG score was 12.6 s (SD 6.5), with men performing better than women; 11.7 s (SD 5.6) versus 13.2 s (SD 7.1) (test for difference between genders: *p* < 0.01) (Table 1, Table 2).

**Table 1:** TUG^a^ in fifths by gender; mean TUG time and number of participants and mortality

	Men		Women	
TUG	N (#dead)	Seconds, mean (SD)	N (#dead)	Seconds, mean (SD)
1 (quickest)	128 (58)	8.2 (1.0)	125 (46)	8.4 (1.2)
2	80 (52)	10.1 (0.3)	138 (52)	10.5 (0.5)
3	69 (38)	11.0 (0.4)	120 (50)	12.4 (0.6)
4	89 (60)	12.8 (1.2)	90 (46)	14.6 (0.7)
5 (slowest)	72 (53)	19.1 (10.3)	94 (51)	23.2 (12.4)
Total	438 (261)	11.7 (5.6)	567 (245)	13.2 (7.1)

^a^Timed Up and Go test

**Table 2:** Background covariates and TUG-score

Variable	N	TUG-score*
Gender		
Men	438	11.7 (5.6)
Women	567	13.2 (7.1)
p-value*		*<0.01*
Age, years		
Men 65–69	14	9.7 (2.2)
Men 70–74	51	11.1 (5.3)
Men 75–79	301	11.4 (4.4)
Men 80+	72	13.8 (9.1)
p-value*		*<0.01*
Women 65–69	11	10.9 (3.8)
Women 70–74	75	11.7 (3.8)
Women 75–79	394	13.6 (7.9)
Women 80+	87	13.2 (4.9)
p-value*		*0.12*
BMI (kg/m^2^)		
< 20	44	12.1 (3.9)
20–24.9	306	11.9 (5.3)
25–26.9	184	12.7 (7.6)
27–29.9	250	12.3 (6.8)
30+	216	13.6 (6.2)
p-value*		*0.09*
Self-reported health		
Poor	39	16.5 (11.9)
Not so good	467	13.8 (7.6)
Good	434	11.0 (3.1)
Very good	25	9.5 (2.3)
p-value*		*<0.01*
Smoking		
Current	160	12.6 (6.8)
Previous	439	12.2 (6.0)
Never	394	13.0 (6.9)
p-value*		*0.96*
Education		
Low	457	12.9 (6.3)
Medium	369	12.1 (5.7)
High	79	11.4 (4.7)
p-value*		*0.18*

*Overall test of association adjusted for age and gender

The age adjusted difference in baseline registration of the TUG score between those who died during follow-up and those who were still alive was 1.5 s (*p* < 0.001). The oldest participants had worse score on TUG compared to younger participants, increasing 0.25 s per year (*p* = 0.001), with similar TUG score decrease with age across genders.

TUG scores were similar across most of the covariates, except for self-reported health (*p*-value <0.01) and BMI (*p*-value: 0.09) (Table 2). Those reporting poor health had a TUG score of 16.5 s and those reporting very good health scored 9.5 s on TUG. Those with BMI at 30 kg/m^2^ or above had the poorest TUG-performance, compared with the other BMI groups.

During follow-up, 506 of the 1005 participants died (50.4%). There was a significant association between TUG-score and mortality; (Table 3). This was the case in both men and women, and the interaction terms gender by TUG-score fifths were non-significant, in both the basically adjusted model and in the fully adjusted model. Across the TUG-score categories, from quickest fifth to slowest fifth, there was a tendency for increased mortality in a step-wise fashion, and especially those in the two poorest TUG performance fifths had high mortality rate; Compared to the quickest fifth, the slowest fifth had hazard ratio (HR) 1.79 (95% confidence interval 1.33, 2.42) in a model adjusted for age and gender. For each SD higher TUG-score the HR was 1.23 (95% CI 1.14, 1.33). The association was robust to further adjustment for BMI, self-reported health status, smoking and education. In such a model the HR for the quickest fifth compared to the slowest fifth was 1.63 (95% CI 1.20, 2.22), and 1.20 (95% CI 1.10, 1.30) per 1 SD increase in TUG-score (Table 3).

**Table 3:** Mortality hazard ratio (HR) by TUG in fifths, by gender, analyzed using Cox regression

	Basically adjusted^a^ (*N* = 864, #deaths = 428)			Fully adjusted^b^ (*N* = 864, #deaths = 428)		
	All	Men	Women	All	Men	Women
TUG	HR (95% CI)	HR (95% CI)	HR (95% CI)	HR (95% CI)	HR (95% CI)	HR (95% CI)
1 (quickest)	1.00	1.00	1.00	1.00	1.00	1.00
2	1.28 (0.96, 1.71)	1.48 (0.99, 2.23)	1.09 (0.71, 1.67)	1.25 (0.93, 1.68)	1.51 (1.00, 2.68)	1.03 (0.66, 1.60)
3	1.23 (0.90, 1.67)	1.25 (0.80, 1.94)	1.18 (0.76, 1.81)	1.16 (0.85, 1.59)	1.22 (0.78, 1.92)	1.05 (0.66, 1.66)
4	1.55 (1.15, 2.09)	1.65 (1.12, 2.44)	1.43 (0.91, 2.25)	1.38 (1.02, 1.87)	1.60 (1.07, 2.39)	1.25 (0.77, 2.02)
5 (slowest)	1.79 (1.33, 2.42)	1.87 (1.25, 2.81)	1.68 (1.08, 2.61)	1.63 (1.20, 2.22)	1.72 (1.13, 2.61)	1.51 (0.95, 2.42)
Per 1 SD increase	1.23 (1.14, 1.33)	1.23 (1.11, 1.37)	1.23 (1.10, 1.37)	1.20 (1.10, 1.30)	1.20 (1.07, 1.34)	1.20 (1.06, 1.36)
Test for trend (across fifths)	*P* < 0.001	*P* = 0.002	*P* = 0.010	*P* = 0.002	*P* = 0.012	*P* = 0.059

^a^Basically adjusted = Adjusted by gender and age; ^b^Fully adjusted = Basically adjusted + adjustment for self-reported health status, smoking and education. (Test for interaction TUG in fifths by gender in basically adjusted model: *p* = 0.835; Test for interaction TUG in fifths by gender in fully adjusted model: *p* = 0.789)

We performed a likelihood ratio test, testing the saturated model containing both TUG and self-reported health against a model without TUG. The saturated model had significantly better fit than the model without TUG (*p* = 0.028). Similarly, testing the saturated model against a model leaving out self-reported health (and keeping TUG in) provided even stronger *p*-value in favor for the saturated model. Hence, self-reported health is a stronger predictor than TUG regarding mortality, but TUG adds predictive value in a model already containing self-reported health. Thus, the association between TUG and mortality is not entirely attenuated by self-reported health.

## Discussion

In this population based study of home dwellers aged 65 years and older followed up over a period of maximum 11.8 years, we found robust evidence of an association between poor TUG score and increased all-cause mortality, which was equally strong in men and women. This association was robust to adjustment for self-reported health, BMI, smoking and education.

To the best of our knowledge, this is the first population based study to examine the association between TUG score and mortality in both genders for subjects aged 65 years or older living at home. Our results of increased mortality for those with poor TUG performance correspond well with results from other similar studies [51, 52, 55–58]. However, some of these studies did not include both genders [51, 52]. The results of De Buyser et al. [51] showed an association of TUG with all-cause mortality in men aged 71–88 years at baseline with 15 years of follow-up. Idland et al. [52] concluded in a study of 300 community-dwelling women aged 75 and older that TUG predicted mortality during 13.5 years-follow up. Furthermore, according to Tice et al. [58] poor results on TUG were strongly associated with increased mortality during 9 years of follow-up in a study of middle-aged community-dwelling postmenopausal women with mean age 68 years.

Our results showed an association of TUG with all-cause mortality in both home-dwelling males and females, not referring to specific disease diagnosis. An example of a study focusing on the association with TUG and mortality in a diagnose specific population is the study of Roshanravan et al. [56] with a sample of 385 participants (mean age 61 years, SD = 13 years) with chronic kidney disease. Their study showed that each one-second longer TUG score was associated with 8% greater risk of death during the following three years, after adjustment for demographics and comorbidity [56]. Robinson et al. [59] found in a prospective cohort study of patients 65 years and older undergoing colorectal and cardiac operation that a preoperative TUG score slower than 15 s was associated with significant higher 1-year mortality. The study of Hoside et al. [56] showed that TUG-score of elderly patients over 80 years of age was associated with total and cardiovascular mortality. Furthermore, TUG was found to predict the risk of death within 6 months in onco-geriatric patients older than 70 years of age receiving chemotherapy [57]. A slower TUG predicts health decline and cognitive decline in community–dwelling older adults [45, 60].

The reasons why TUG is predictive of mortality might reflect underlying malaise as well as chronic illness. Despite, the TUG test has been proposed as a single measure to identify older adults who are at high risk for adverse health outcomes [61]. In older adults, the ability of the TUG test to capture comorbidity burden highlights the importance of the multiple interactions among several different body systems (e.g. nervous, cardiopulmonary, and musculoskeletal systems) involved in coordinating mobility and balance. For example, slower gait speed has been associated with subclinical cerebrovascular disease, even among apparently high functioning older adults [62–65]. Walking speed may capture both underlying age-related biological changes, as well as diagnosed and undiagnosed diseases [66]. Furthermore, it may reflect an individual’s vitality because walking requires energy and puts demand on body functions and structures [66]. Above-average physical performance probably reflects a resilience that enables a person to respond adequately to future stressors [67]. In fact walking speed is an important component in TUG [68].

As in other studies where data were analyzed by gender we found that men had significantly better TUG score than women [69–71], and that TUG was positively associated with age [70, 72]. However, we found no gender- specific association between TUG and mortality in our study. Hence, TUG seems to predict mortality equally well in men and women.

Non-participants tend to have higher mortality than participants in population-based studies [73], and if this was the case in our study and non-participants also were more frail than the participants, this may have underestimated the true relationship between TUG and mortality. Hence, our estimate might be a conservative one. Our study had an age range from 65 and above, but the majority were in the age group 75–79 (69%), and age specific results are therefore most robust in this age group. All observational studies are hampered by residual confounding to various degree. We included key health related confounders (self-reported health, smoking, education) which only to a limited degree attenuated the TUG-mortality association. However, mild cognitive impairment and early stage dementia may bias results since there is strong evidence of a consistent association between cognitive function and different aspects of physical fitness related to independent living such as gait speed, mobility, balance and muscle strength [74, 75]. The strength of the present study includes long follow-up time, objective measures of physical capability and the inclusion of key covariates. Death certificates were obtained from the official Cause of Death Register, which covers the whole population, and is of high quality.

## Conclusions

The present study may add new knowledge to the limited research on gender specific analyses on the association between mobility and mortality in a general population, by showing that TUG score is an equally important predictor for survival in both men and women. Identifying older people with poor TUG score may aid in identifying those at risk and thus targeted interventions may be applied.

## Abbreviations

BMI: Body mass index
CI: Confidence interval
HR: Hazard ratio
SD: Standard deviation
TUG: Timed up and go
